# Care-seeking behaviour among febrile children under five in Togo

**DOI:** 10.1186/s12889-022-14550-6

**Published:** 2022-11-17

**Authors:** Gountante Kombate, Gbènonminvo Enoch Cakpo, Komi Ameko Azianu, Matè Alonyenyo Labité, Marianne A. B. van der Sande

**Affiliations:** 1 Society for Study and Research in Public Health, Ouagadougou, Burkina Faso; 2 Institut Supérieur Des Sciences de La Population, Ouagadougou, Burkina Faso; 3 Global Health, Julius Centre, University Medical Centre Utrecht, Utrecht, The Netherlands; 4 Department of Public Health, Institute of Tropical Medicine, Antwerp, Belgium

**Keywords:** Fever, Care-seeking, Children under five, Togo

## Abstract

**Background:**

Fever is one of the warning signs of poor health in children. Care-seeking in febrile children is importance in reducing child deaths and morbidity. This care-seeking by parents in children with fever is however relatively low in sub-Sahara Africa. The aim of this study is to improve understanding of the behaviour of caregivers in seeking care for children under five with fever and to identify associated modifiable risk factors in Togo.

**Methods:**

Data from a 2013–2014 cross-sectional nationally representative malaria indicator survey was used. Advice or care-seeking is defined as any child under 5 years of age with fever in the two weeks prior to the interview for whom advice or treatment was sought in a public medical area, private medical area, store, market, or from an itinerant medicine seller. Univariate and multivariate logistic regression analysis were performed using Generalized Linear Models.

**Results:**

A total of 1359 febrile children out of 6529 children under five were enrolled. Care had been sought in 38.9% of cases. In multivariate analysis, independent risk factors associated with formal care seeking were accessibility to the nearest health center (aOR = 1.52, 95% CI [1.18–1.95], mother's education level secondary and above (aOR = 1.85, 95% [1.32–2.59]), mothers who identified as belonging to animist/traditionalist religions compared to mothers who belonged to a formal religion (catholic (aOR = 2. 28, 95% [1.55–3.37]), Muslim (aOR = 2.41, 95% [1.67–3.47]), and Protestant (aOR = 1.9, 95% [1.37–2.65]), Maritime region (aOR = 0.49, 95% [0.29–0.82]) compared to Lome commune.

**Conclusion:**

Interventions should specifically target women with limited education, not identifying as part of an official church and at longer distance from health center.

## Introduction

Fever is one of the main signs of illness in children [1] and the primary reason for paediatric health centre consultations and hospitalisations in Africa [2]. Manifested by an elevation in the temperature of the body [3] fever is generally suggestive of an infectious disease, including pneumonia and malaria, which are the main causes of death in children under five in sub-Saharan Africa in 2022 [4]. Over the past two decades, there has been considerable progress in reducing child mortality. A decline from 93 deaths per 1,000 live births in 1990 to 37 per 1,000 live births in 2020 has been recorded [5]. Despite this progress, improving child survival remains a public health priority. In 2020 alone, more than 5 million children died before reaching the age of five in sub-Saharan Africa. Children in sub-Saharan Africa and South Asia bear the greatest burden of child mortality [5]. A large proportion of these deaths were due to preventable and treatable diseases [6, 7].

Several factors may result in care not being sought, including distance to health centers, lack of resources including staff, perceived poor quality, costs, etc. If care is being sought, it may not always be appropriate or timely, for reasons including poor quality of care, stock-outs and inadequate health staff training [8]. Other factors such as socio-demographic factors, socio-cultural factors, socio-economic factors, and environmental factors could also contribute to care not being sought in time [4, 9].

In Togo, the infant and child mortality rate was 64 deaths per 1,000 live births in 2020 [10]. Nationally, of all children reported with a fever, for 24% care was sought in 2014 and 56% in 2017 [11]. Very few studies have addressed care-seeking behaviour among febrile children in Togo [12, 13]. Such studies showed that seeking care from a health provider was not frequent while self-medication and reliance on traditional healers during illnesses remain common practice with 85.8% [12] and 80.2% [13] according to these two studies in Togo. So far, the few studies conducted in Togo have included small samples that did not allow extrapolation towards policy adjustments at national level. The present study was conducted in this context with the objective to improve understanding of the behaviour of caregivers in seeking care for children under five with fever and to identify associated modifiable risk factors, using national population-based data [14].

### Method

#### Background to the study

**Fig. 1:**
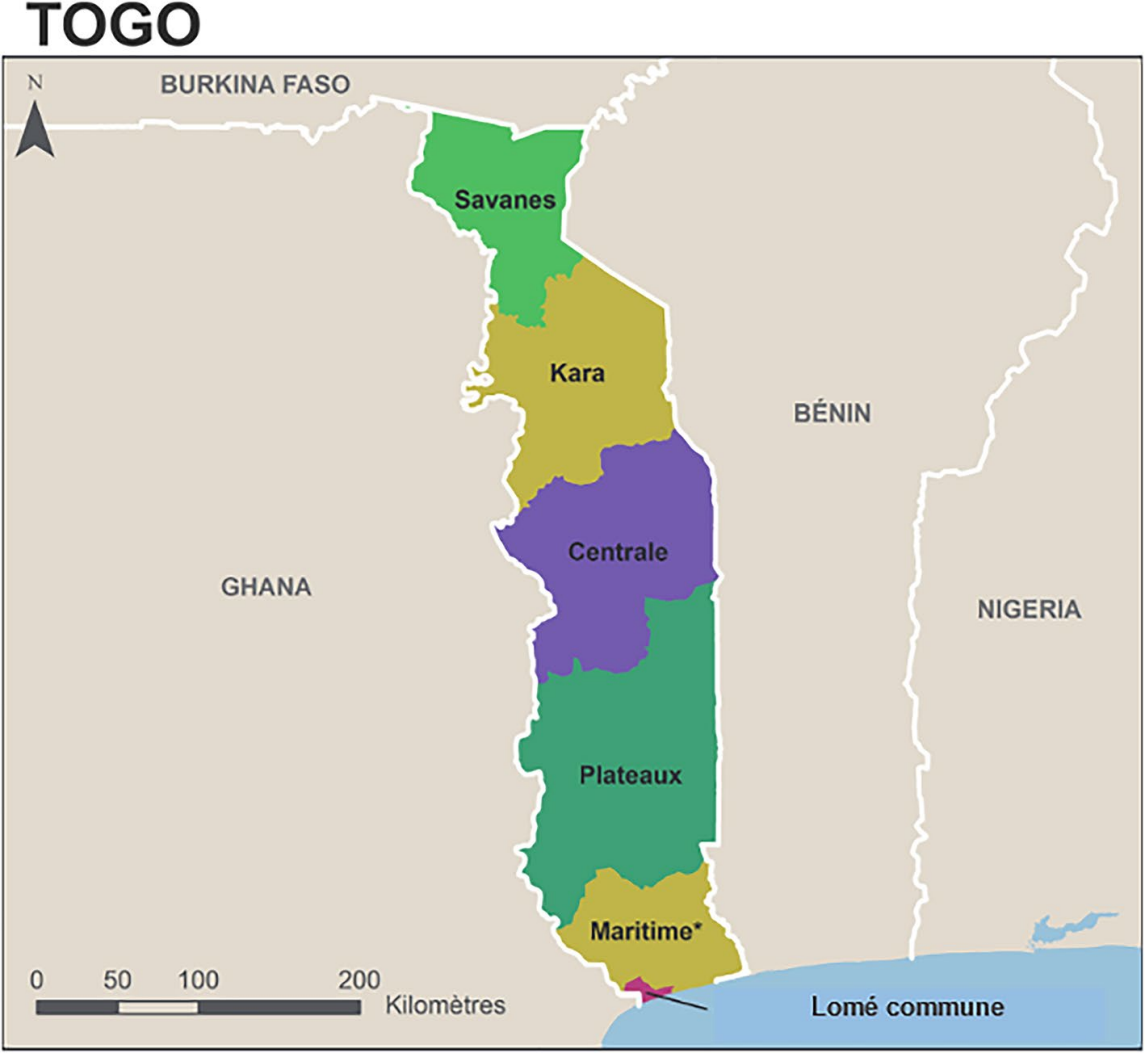
Togo's administrative map (Ministry of Health, 2018)

Togo is located in West Africa, with a population that has grown from 2,719,567 in 1981 to 7,886,000 and a density of 152 inhabitants/km2 in 2021 [15]. It is bordered to the north by Burkina Faso, to the south by the Gulf of Guinea, to the east by Benin and to the west by Ghana. It has six health regions and forty-three health districts in total (Fig. 1).

The country has a tropical climate (hot and humid), which is partly responsible for the national epidemiological disease profile dominated by infectious and parasitic diseases [16]. The health system in Togo is organised according to a three-tier pyramid structure [17]. The first level consists of the central administration and the various departments and programmes where national directives and policies are developed. The regional (or intermediate) level comprises six health regions that provide coordination and technical support to the third-level health districts. The peripheral level is represented by the health district, which is the most decentralised operational entity, comprising 43 health districts and 944 peripheral health units [17].

## Type of study and sampling

Data were used from the Togo Demographic and Health Survey (TDHS) 2013–2014 [18]. TDHS (2013–2014) included a representative probability sample of 9,899 households (3,840 in urban areas in 128 clusters and 6,059 in rural areas in 202 clusters). Sample was based on a stratified, two-stage area survey. All women aged 15–49 usually living in the selected households or present the night before the interview with or without children under five were eligible to be interviewed. A total of 9,480 women aged 15–49 (3,591 in urban areas and 5,886 in rural areas) and 6,529 children aged 0–5 years were included in the survey [18]. The study population consisted of children under five years of age for whom fever was reported in the two weeks prior to the survey.

## Tools and data collection

Three questionnaires were used in the context of the TDHS 2013–2014 (a household questionnaire, a women's questionnaire, and a biomarker questionnaire). The household questionnaire recorded all household members and visitors who slept the night before the interviewer visited the household. The women's questionnaire was used to collect information on socio-demographic characteristics, knowledge of malaria, births over the last 5 years, prevalence and treatment of fever in children under five, exposure to malaria prevention measures. Information on childhood illness and health care-seeking behaviour during the two weeks preceding the survey was requested. Our study included children under the age of five that had a fever two weeks prior to the surveys. They were children who had a hot body or had a temperature higher than 37.5 degrees. Data collection took place between 10 November 2013 to 15 April 2014.

### Study variables

The dependent variable was seeking formal care (Yes: if advice or care was sought from a health professional in public hospitals, dispensaries, and private clinics including community health workers; No: if advice or care was sought from itinerant sellers, pharmacies, shops, markets, families, friends, traditional healers or no advice at all) [4, 19, 20]. The independent variables were the age and sex of the child, whether the child was breastfeeding at the time of the survey, the age of the woman, her level of education, the area of residence, the region, the distance to the nearest health centre, the marital status, the wealth index of the household, the profession, the religion, the ethnicity and the sex of the head of household. The wealth index was constructed through principal component analysis using information on household assets including the possession of a number of consumer goods and housing characteristics [21, 22].

### Data analysis

For each of the recoded variables, we assessed the associations with the dependent variable by performing a univariate logistic regression using generalized linear models (GLM) and calculated the Odds ratios. Any association that was found statistically significant at a level of *p* ≤ 0.10 in the univariate analysis was included in a multivariate model. We then removed one by one, starting with the highest *p*-value, potential confounding factors and checked whether this resulted in a change of more than 10% in the Odds Ratio and/or whether the likelihood ratio test was significant (*p* ≤ 0.05). If these conditions were met, the potential confounding factor was retained otherwise it was removed from the model. This process was continued until all remaining potential confounders were significant as confounders. With the variables retained (*p* ≤ 0.05), we tested for interactions between our dependent variable and each of the variables retained in the final model [23]. Data were analysed using R software version 4.0.4.

## Results

In total, the survey identified 1359 febrile children out of 6529 children under five (20.8%). There were more febrile boys (51.1%) than girls (48.9%), and the median age of the febrile children was 28 months (IQR 16–42). The largest group of children in the sample (28.7%) were from the poorest wealth index, while only 12.4% were from the richest wealth index. The majority (71.3%) lived in rural areas (Table 1). Among these children, care had been sought in 38.9% of cases.

**Table 1:** Study population (*n* = 1359)

Children who had fever in the two weeks before the survey	
	N (%)
Age (months)	
0–11	221 (16.3)
12–23	359 (26.4)
24–35	289 (21.3)
36–47	269 (19.7)
48–59	221 (16.3)
Sex	
Male	694 (51.1)
Female	665 (48.9)
Household head's sex	
Male	1151 (84.7)
Female	208 (15.3)
Currently breastfeeding	
No	579 (42.6)
Yes	780 (57.4)
Place of residence	
Urban	391 (28.7)
Rural	968 (71.3)
Distance to the nearest health	
Big problem	479 (35.2)
Not a big problem	880 (64.8)
Region	
Lome commune	256 (18.9)
Maritime	204 (15.1)
Plateaux	420 (30.0)
Centrale	126 (09.2)
Kara	134 (09.8)
Savanes	219 (17.0)
Age of caregiver	
15–24	305 (22.4)
25–34	682 (50.2)
35–49	372 (27.4)
Caregivers' level of education	
No education	612 (45.1)
Primary	472 (34.7)
Secondary and high	275 (20.2)
Wealth index	
Poorest	339 (25.0)
Poorer	314 (23.1)
Middle	279 (20.5)
Richer	238 (17.5)
Richest	189 (13.9)
Marital status	
Married	1251 (92.1)
Divorced/widowed	63 (04.6)
Single	45 (03.3)
Occupation	
Farmer/trader	960 (70.6)
Government worker	220 (16.2)
Not working	179 (13.2)
Head of household's religion	
Animist/traditionalist	451 (33.2)
Catholic	251 (18.4)
Muslim	213 (15.6)
Protestant and others christian	444 (32.8)
Ethnicity	
Ewe/mina/ana-ife	511 (37.6)
Kabye/tem	331 (24.3)
Paragroma/akposso/akebou	452 (33.3)
Others	65 (04.8)

### Care-seeking and associated factors

Table 2 summarises the bivariate and multivariate results for care-seeking among children with fever in the two weeks preceding the survey.

**Table 2:** Potential factors associated with advice or care seeking for febrile children in Togo (*n* = 1359) DHS 2013

	**Advice or health care seeking**			
Variables	Yes (%)	No (%)	Crude OR (95% CI)	Adjusted OR (95% CI)
Age				
0–11	91 (41.2)	130 (58.8)	Ref	-
12- 23	137 (38.2)	222 (62.8)	0.81 (0.57–1.13)	-
24–35	107 (37.0)	182 (63.0)	0.80 (0.56–1.14)	-
36–47	111 (41.3)	158 (58.7)	0.87 (0.62–1.24)	-
48–59	82 (37.1)	139 (62.9)	0.78 (0.54–1.13)	-
Child sex				
Male	266 (38.3)	428 (61.7)	Ref	-
Female	262 (39.4)	403 (60.6)	0.98 (0.79–1.21)	-
Household head's sex				
Male	446 (38.7)	705 (61.3)	Ref	-
Female	82 (39.4)	126 (60.6)	1.04 (0.76–1.42)	-
Currently breastfeeding				
No	233 (40.2)	346 (59.8)	Ref	-
Yes	296 (37.9)	484 (62.1)	0.91 (0.56–0.93)	-
Place of residence				
Urban	190 (48.6)	201 (51.4)	Ref	Ref
Rural	338 (34.9)	630 (65.1)	0.71 (0.56–0.93)	1.73 (0.94–3.24)
Accessibility to nearest health centre				
Problematic	153 (32.1)	326 (67.9)	Ref	Ref
Not problematic	375 (42.6)	505 (57.4)	1.51 (1.21–1.89)	1.52 (1.18–1.95)
Region				
Lomé commune	126 (49.2)	130 (50.8)	Ref	Ref
Maritime	59 (28.9)	145 (71.1)	0.34 (0.20–0.55)	0.49 (0.29–0.82)
Plateaux	131 (31.2)	289 (68.8)	0.59 (0.41–0.86)	0.79 (0.54–1.16)
Centrale	49 (38.9)	77 (61.1)	0.78 (0.52–1.18)	0.84 (0.55–1.29)
Kara	64 (47.8)	70 (52.2)	1.11 (0.74–1.67)	1.64 (0.90–2.54)
Savanes	99 (45.2)	120 (54.8)	1.06 (0.76–1.49)	1.80 (0.99–2.74)
Women's age				
15–24 yrs	123 (40.3)	182 (59.7)	Ref	-
25–34 yrs	272 (39.9)	425 (60.1)	0.99 (0.51–0.81)	-
35–49 yrs	133 (35.8)	262 (64.2)	0.78 (0.57–1.06)	-
Education level				
No education	201 (32.7)	411 (62.1)	Ref	Ref
Primary	188 (40.0)	283 (64.8)	1.12 (0.88–1.44)	1.15 (0.87–1.52
Secondary and	139 (47.3)	137 (52.7)	1.91 (1.43–2.55)	1.85 (1.32–2.59)
higher Wealth Index				
Poorest	123 (36.3)	216 (63.7)	Ref	Ref
Poorer	99 (31.4)	215 (68.6)	0.82 (0.61–1.11)	1.02 (0.73–1.42)
Middle	95 (34.0)	184 (66.0)	0.85 (0.62–1.17)	1.07 (0.74–1.55)
Richer	104 (43.8)	134 (56.2)	1.30 (0.92–1.82)	1.56 (0.99–2.44)
Richest	107 (56.1)	82 (43.9)	1.62 (1.12–2.33)	1.92 (0.99–3.35)
Marital status				
Married	483 (38.6)	768 (61.4)	Ref	-
Divorced/widowed	29 (46.0)	34 (54.0)	0.82 (0.50–1.34)	-
Single	16 (35.6)	29 (64.4)	0.81 (0.36–1.75)	-
Occupation				
Farmers/traders	358 (37.3)	602 (62.7)	Ref	-
Public employee	71 (32.3)	149 (67.7)	1.34 (0.96 -1.87)	-
Not working	99 (55.3)	80 (44.7)	1.10 (0.65–1.83)	-
Religion				
Animist/Traditionalist	101 (22.4)	350 (77.6)	Ref	Ref
Catholic	115 (45.8)	136 (54.2)	2.20 (0.29–0.47)	2.28 (1.55–3.37)
Muslim	119 (55.9)	94 (44.1)	2.31 (1.65–3.23)	2.41 (1.67–3.47)
Protestant	193 (43.5)	251 (56.5)	1.57 (1.17–2.10)	1.90 (1.37–2.65)
Ethnicity				
Ewe/mina/ana-ife	133 (26.0)	378 (74.0)	Ref	Ref
Kabye/tem	125 (37.8)	206 (62.2)	1.16 (0.86–1.56)	0.90 (0.61–1.32)
Paragrouma/akposso/akebou	246 (54.4)	206 (45.6)	1.58 (1.21–2.06)	1.14 (0.78–1.65)
Others	24 (36.9)	41 (63.1)	2.55 (1.38–4.77)	1.61 (0.76–3.41)

*OR* Odd ratio, *Ref* Reference modality, *95% CI* 95% Confident Interval

Care was sought more for children living in urban areas (48.6%) compared to those living in rural areas (34.9%). Women with secondary and higher education more often (47.3%) looked for care, compared to those with primary education (40.0%) and no education (32.7%). For febrile children living in the Maritimes region care was sought less (28.9%) compared to those living in the Lomé commune region (49.2%). However, it should be noted that 39.1% of caregivers did not seek advice or care from any source.

After bivariate analyses, residence, region, distance to the nearest health centre, maternal education, wealth index, ethnicity and region were found strongly and significantly associated with seeking advice or care. No association was observed with the sex of the child and the sex of the head of household. In multivariate analyses, the final predictors of formal advice or care-seeking was distance to the nearest health centre, region, mother's education, and religion. Febrile children whose mothers perceived easy access to the health centre were more likely (OR = 1.52 (1.18–1.95)) to access care compared to those whose mothers perceived difficult access. Mothers who identified as belonging to animist/traditionalist religions were less likely to use care compared to mothers who belonged to a formal religion. The results also showed that women with a secondary education and above were more likely (OR = 1.85 (1.32–2.59)) to seek formal advice or care compared to those with no education. This relationship showed a clear dose–response curve such that for each increase in the mother's education level, the likelihood of seeking advice or care for her child with fever increased.

Caretakers living in the Maritime region remained less likely (OR = 0.49 (0.29–0.82)) to seek advice or care compared to those living in other parts of the country.

## Discussion

Care seeking for under 5 febrile illness was strongly associated with region, mother’s education, religion and distance to nearest facility. The current study used data from a nationally representative household survey and the results can therefore be considered representative for the national level. However, it should be noted that these data are based on self-reporting of fever by mothers of children and are not validated by medical examination. The completeness of reporting by mothers of children is subject to recall bias which could vary from mother to mother. Also, information on fever characteristics such as severity, duration, location of fever, and perceived quality of care was not collected. This missing information could be relevant factors determining care seeking or not in case of fever in children.

Care was sought for only 38.9% of all children with reported fever in the two weeks preceding the survey. Similar results were found in Malawi [24, 25], Nairobi [26], Burkina Faso [4], and Nigeria [27]. This low uptake of care could be explained by an inability of mothers to recognise fever as a danger sign for the child, cultural aspects, time constraints, lack or cost of transport to the nearest health centre. One of the surprising results is that wealth index was not significantly associated with formal advice or care seeking behaviour. One of the main explanations could be the free of charge child care policy introduced in Togo in 2012. This result is contrary to those obtained in Burkina Faso [4]; and Mozambique [28]. For its authors, despite the different policies of free of charge child health care instituted in these countries, care-seeking by parents in children with fever remained relatively low [4, 28]. Also, a study in Mozambique [29] found that the quality of care, unavailability or stock-outs of effective treatment, and long waiting lines are factors that may discourage households from seeking care in health facilities despite the policies of free access.

Maternal education increased the probability of seeking care. Similar results have been reported in Malawi [24] and Nigeria [27]. Maternal education definitely improves understanding of the disease and the ability to seek formal advice or care. These findings reinforce the need to improve literacy rates in Togo as a means to improve care-seeking behaviour. Mothers living in the Maritime region were less likely to seek advice or care compared to those living in the rest of the country. Similar findings were also reported in other studies in Burkina Faso [4] and Malawi [19]. This regional difference is probably related to cultural differences in the perception of health and illness [19]. A study in Uganda, reported similar regional variations in health care seeking behaviour for children [24, 27]. Religion was also strongly associated with seeking advice or care. Compared to the traditionalists, mothers who were Christian and Muslim were more likely to seek formal advice or care in case of fever in their children. This could be explained by the fact that traditionalists more often use plants and tree bark as the first line of care. [27, 30].

The above results have improved the understanding of care-seeking behaviour of parents of children with fever illness. These data are currently the best data available, especially in a Togolese context, and action is needed. Accessibility to care has been a strongly associated and significant factor in the use of care and needs to be explored in advance in the context of the policy of free health care for children under the age of five in Togo.

## Conclusion

The study identified factors associated with seeking care among children under five with fever two weeks preceding the survey. The results confirmed that care seeking for fever in public and private health facilities in Togo is still suboptimal. Geographic variation at the regional level was identified. Interventions to improve universal primary health care coverage in terms of geographical accessibility, literacy and health education are to be encouraged. The regional differences highlighted are additional considerations for such interventions [31]. Qualitative studies could provide a further understanding of advice or care-seeking behaviour for fever among children in Togo.

## Data Availability

DHS datasets are publicly available on www.dhsprogram.org

## Abbreviations

aOR: Adjusted Odds ratios
BCRS: Bioethics Committee for Health Research of Togo
DHS program: Demographic and Health Survey program
GLM: Generalized Linear Models
IQR: Inter-quartile range
MoH: Ministry of Health
OR: Odds ratios
TDHS: Togo Demographic and Health Survey
